# CRISPR-activation screen identified potassium channels for protection against mycotoxins through cell cycle progression and mitochondrial function

**DOI:** 10.15698/cst2023.05.279

**Published:** 2023-04-18

**Authors:** Yulong Tang, Simeng Liao, Zhuyuan Nie, Guangwei Kuang, Chunxiao Ji, Dan Wan, Liuqin He, Fengna Li, Xiangfeng Kong, Kai Zhan, Bie Tan, Xin Wu, Yulong Yin

**Affiliations:** 1 Laboratory of Animal Nutritional Physiology and Metabolic Process, Key Laboratory of Agro-ecological Processes in Subtropical Region, National Engineering Laboratory for Pollution Control and Waste Utilization in Livestock and Poultry Production, Institute of Subtropical Agriculture, Chinese Academy of Sciences, Changsha 410125, China.; 2 College of Animal Science and Technology, Hunan Agricultural University, Changsha, China.; 3 Hunan Provincial Institute of Animal Drug and Feed Supervision, Changsha, 410006, China.; 4 Anhui Province Key Laboratory of Livestock and Poultry Product Safety Engineering, Hefei, 230001, China.

**Keywords:** CRISPR-Cas9 screening, zearalenone, potassium channels, cell cycle, mitochondria

## Abstract

Zearalenone (ZEA) exposure has carcinogenic effects on human and animal health by exhibiting intestinal, hepatic, and renal toxicity. At present, the underlying mechanisms on how ZEA induces apoptosis and damage to tissues still remain unclear. In this study, we aimed to identify genes that modulate the cellular response to ZEA using clustered regularly interspaced short palindromic repeats (CRISPR)-Cas9 screening, and further validate novel gene functions to elucidate molecular mechanisms underlying particular biological processes *in vivo* and *in vitro*. Two ZEA-resistant cell lines, designated Ov-KCNJ4 and Ov-KCNJ12, were yielded by CRISPR activation screening which had significant changes in ZEA resistance and growth rates. Results showed that ZEA could interact with the cell membrane proteins *KCNJ4* and *KCNJ12*, inducing cell cycle arrest, disruption of DNA replication and base excision repair. Overexpression of *KCNJ4* and *KCNJ12* was involved in ZEA resistance by regulating cell cycle to neutralize toxicity, sustaining mitochondrial morphology and function via attenuating the damage from oxidative stress in the KCNJ4-mitoK_ATP_ pathway. *In vivo* experiments showed that AAV-KCNJ4 delivery significantly improved ZEA-induced renal impairment and increased antioxidative enzyme activity by improving mitochondrial function. Our findings suggest that increasing potassium channel levels may be a putative therapeutic target for mycotoxin-induced damage.

## INTRODUCTION

CRISPR-based genome-scale screening has facilitated the identification of functional phenotypes in a high-throughput setting [1–4]. Generally, CRISPR-Cas9 can mediate all cell genes by yielding knockout or activation pooled libraries and the desired phenotypes by positive- or negative-selection strategies. Therefore, the genetic features involved in the phenotypic change can be identified and validated. Currently, pooled genetic screens based on CRISPR technologies act as invaluable tools for the identification of cell-essential genes, drug resistance mediators, relevant receptors, and toxin metabolism genes, which contribute to elucidating unknown mechanisms in biology [5, 6].

Zearalenone (ZEA), also known as RAL or F-2 toxin, is a potent estrogenic mycotoxin produced by *Fusarium* and *Gibberella* [7, 8]. Humans are exposed to ZEA directly by contaminated food or indirectly through products derived from animals infected with mycotoxins. After ingestion, ZEA and its metabolites accumulate in various tissues and subsequently affect human and animal health, such as inducing necrosis of the intestinal epithelium, disrupting intestinal barrier function, and destruction of immune responses [9]. Moreover, ZEA has been reported to have carcinogenic effects on mammalian species. Extensive work with human cancer cell lines has shown that ZEA and some of its metabolites play important roles in the stimulation of hormone-dependent tumors, particularly for those arising in the breast and endometrium [8, 10, 11]. Additionally, ZEA and its metabolites were present in nearly all of the surveyed women and its concentration was dependent on meat intake as well as body mass index. Epidemiological studies on the potential association between urinary concentrations of ZEA and its metabolites and breast cancer suggested a potential role in the risk of developing breast cancer [8]. Although ZEA is known to induce apoptosis and cell damage by increasing levels of reactive oxygen species (ROS) [9], the underlying mechanisms still remain unclear.

Ion channels are transmembrane proteins which form cellular pores, drive the selective flow of ions, and are involved in maintaining electrochemical gradients across the cell membrane [11]. Potassium ion channels can control the duration of both resting and action potentials and regulate a variety of cellular functions, including mitotic biochemical signaling, cellular differentiation and volume regulation, and cell proliferation [13–15]. We observed that overexpression of potassium channels can lead to resistance to toxins. In order to confirm the relationship between ZEA and potassium channels, multiple cells were edited *in vitro* or *in vivo* to reveal the underlying mechanisms of two genes conferring ZEA resistance. Besides, we also provided evidence of cell survival being facilitated by potassium channel genes following mycotoxin exposure.

## RESULTS

### Positive-selection CRISPR screening using ZEA toxin

**Figure 1:**
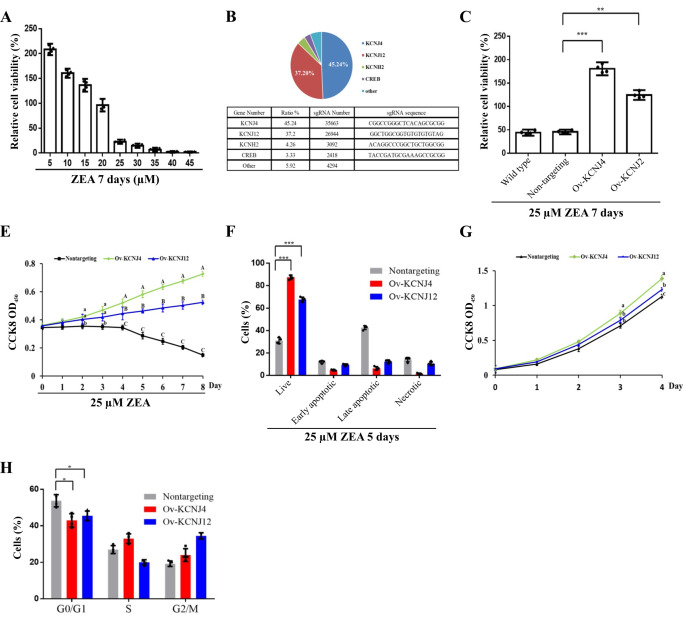
FIGURE 1: CRISPR-Cas9 screening generated two ZEA resistant intestinal cell lines. **(A)** The viability of HCT-8 cells was assessed following treatment with varying concentrations of ZEA for seven days. **(B)** Top four genes in the CRISPR-Cas9-based screen following treatment with 25 µM ZEA. **(C)** Cellular viability was determined for wild type, non-targeting-sgRNA-transduced cells, overexpression of *KCNJ4* and *KCNJ12* for seven days. **(D)** The three cell lines with the indicated tags were counted daily to assess their survival after treatment with 25 µM ZEA. **(E)** Percentages of necrosis, survival, and apoptosis were measured by PI/AnnexinV staining and flow cytometry. **(F)** The three cell lines were counted daily to assess their proliferation ability. **(G)** Cell cycle progression of the three cell lines was assessed. Data of figure D and figure F are expressed as means ± SEM. (n = 4/group). A two-way ANOVA with Bonferroni posttest was used for Figures D and F. A, B, C Values within a row with different superscripts differ significantly (P < 0.05); A, B, C Values within a row with different superscripts differ significant (P < 0.01). Other Data are presented as the mean ± SEM (n=4). p < 0.05 (*), p < 0.01 (**) and p < 0.001 (***), calculated by Student t-test.

To identify the mechanisms of ZEA toxin-induced cell death, a CRISPR-Cas9-based positive-selection was conducted to screen for target genes. Genes were selected whose transcriptional activation permitted cell survival and proliferation in the presence of 25 µM ZEA, a concentration that severely disrupts cell viability (**Fig. 1A**). HCT-8 human epithelial cells were transduced with lentiviral libraries (Addgene #1000000078) in the presence of ZEA for approximately two weeks. Single guide RNA (sgRNA) sequences from the surviving cells were identified via next-generation sequencing (NGS) yielding81733 total reads. The candidate genes were ranked based on the number of unique sgRNAs and NGS reads (**Fig. 1B**).

The screen revealed more than five gene hits, two of which covered 82.44% of reads with 67380 NGS reads. Both *KCNJ4* and *KCNJ12* belong to the inward rectifier potassium channel family, which have been linked with cancer progression and might be valuable prognostic biomarkers and potential therapeutic targets for cancers [15, 16]. To further confirm the involvement of these two genes in ZEA resistance, two overexpression cell lines (Ov-KCNJ4 and Ov-KCNJ12) were created by cloning the cDNA sequence into the pLVX-puro plasmid. Subsequently, the resistance of these genes was evaluated. Under ZEA exposure for eight days, both overexpression cell lines exhibited significant resistance to ZEA and maintained the ability to grow while most of the nontargeting cells could not withstand ZEA toxicity and died (**Fig. 1C, D, E**). In addition, the increased expression of the two genes promoted proliferation ability and cell cycle progression under normal conditions (**Fig. 1F, G**). Taken together, these results indicate that the overexpression of the *KCNJ4* and *KCNJ12* genes could generate ZEA resistant cells.

### Knockout cell lines of *KCNJ4* and *KCNJ12* genes render sensitivity to ZEA toxin

**Figure 2:**
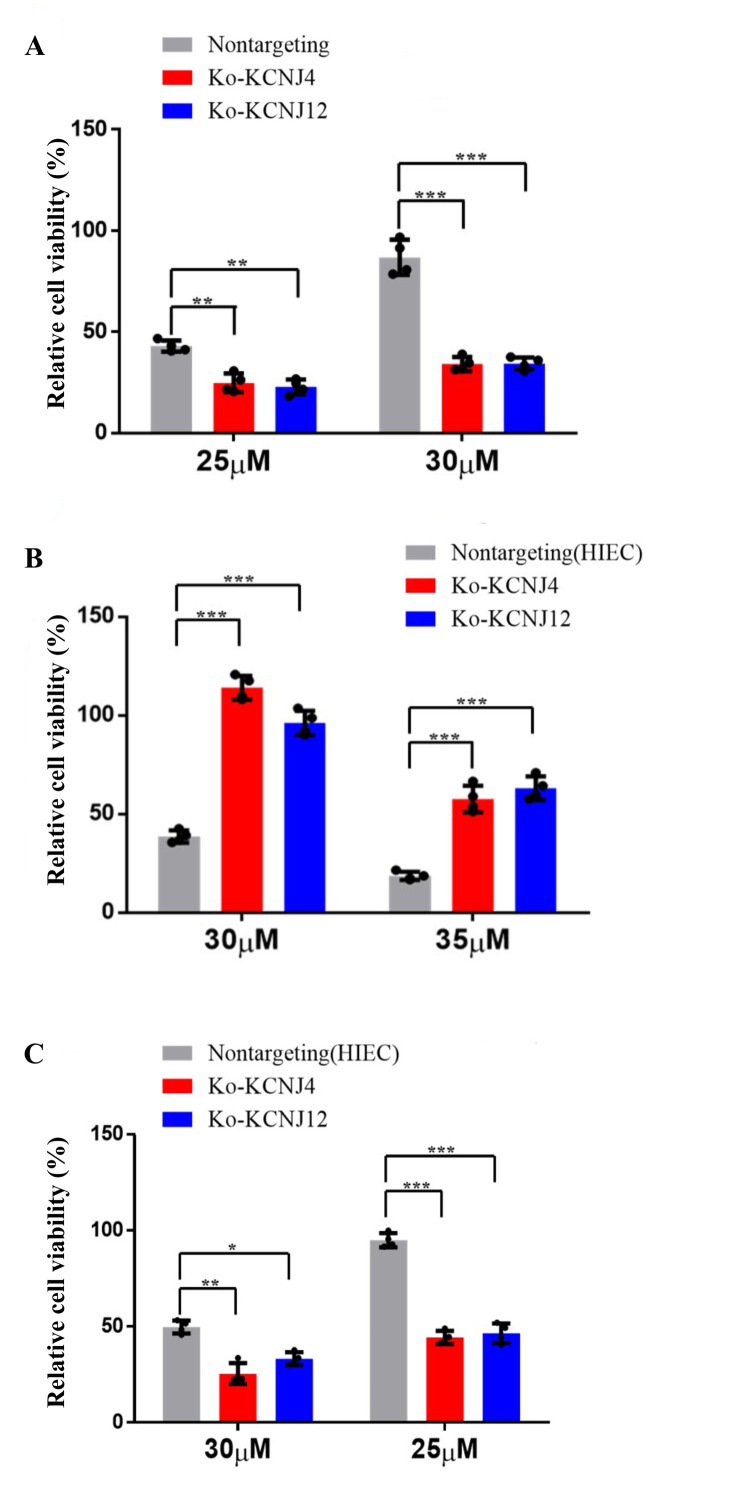
FIGURE 2: Overexpression and knockout of *KCNJ4* and *KCNJ12* regulates ZEA toxin resistance. **(A)** Knockout of *KCNJ4* and *KCNJ12* and the percentage cellular viability of cell lines after treatment with either 20 or 25 µM of ZEA. **(B)** Overexpression of *KCNJ4* and *KCNJ12* in another cell line (HIEC) and the percentage cellular viability after treatment with either 30 or 35 µM of ZEA. **(C)** Knockout of *KCNJ4* and *KCNJ12* in another cell line (HIEC) and the percentage cellular viability after treatment with either 30 or 35 µM of ZEA. Data are presented as the mean ± SEM (n=4). p < 0.05 (*), p < 0.01 (**) and p < 0.001 (***), calculated by Student t-test.

Potassium channel proteins participate in several important features in mammalian cell physiology. They are the targets of multiple toxins and the malfunction of potassium channels is associated with numerous diseases. To further determine whether potassium channel proteins were responsible for conferring ZEA resistance, knockout cell lines of *KCNJ4* and *KCNJ12* were created. Compared to parental cells, both knockout cell lines showed increased sensitivity to ZEA exposure. As shown in **Fig. 2A** the decrement of ZEA concentration resulted in a significant reduction in the survival rates in the knockout cell lines compared to the control group. In addition, we also validated that the overexpression or deletion of *KCNJ4* and *KCNJ12* significantly enhanced or weakened intestinal cell line (HIEC) cell viability upon exposure to ZEA (**Fig. 2B, C**).

### ZEA affects cell cycle progression and causes cell damage by targeting the potassium channels in early stages of ZEA exposure

**Figure 3:**
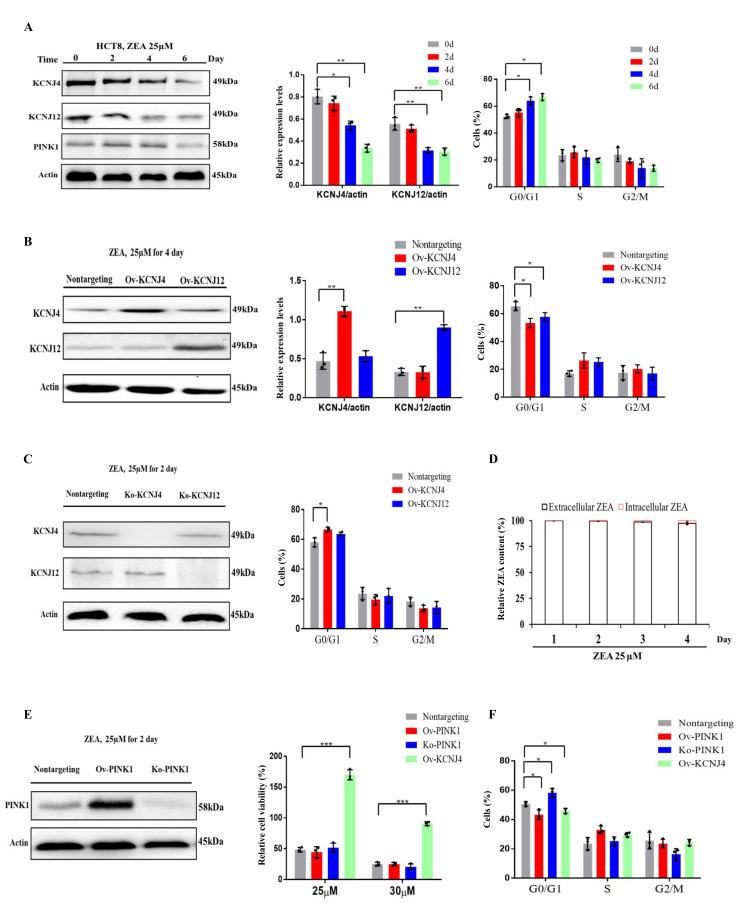
FIGURE 3: ZEA blocks cell cycle progression through targeting the potassium channels in the early stages of ZEA treatment. **(A)** Immunoblotting for KCNJ4, KCNJ12, PINK1 and actin expression after ZEA treatment as well as assessment of cell cycle progression in HCT-8. **(B)** Immunoblotting for KCNJ4, KCNJ12 and actin expression after ZEA treatment as well as assessment of cell cycle progression in overexpression cell lines. **(C)** Immunoblotting for KCNJ4, KCNJ12 and actin expression after ZEA treatment as well as their cell cycle in knockout cell lines. **(D)** ZEA toxin relative concentrations (extracellular and intracellular) in HCT-8 cells exposed to ZEA toxin. **(E)** Knockout and overexpression of *PINK1* and the percentage cellular viability of cell lines after treatment with either 25 or 30 µM of ZEA. **(F)** Knockout and overexpression of *PINK1* and assessment of cell cycle progression. Data are presented as the mean ± SEM (n=3). p < 0.05 (*), p < 0.01 (**) and p < 0.001 (***), calculated by Student t-test.

The transcriptome results showed that the earliest toxin-induced biochemical alterations could hamper cell proliferation and cell cycle progression when the cells were not damaged (Fig. S1 A, B). Two genes, *KCNJ4* and *KCNJ12*, that play an important role in controlling cell division and cell cycle and belong to voltage-gated potassium channels, were screened. Results indicated that ZEA exposure significantly suppressed the expression of *KCNJ4* and *KCNJ12* accompanied by the inhibition of cellular proliferation and cell cycle progression (**Fig. 3A** and Fig. S2). Overexpression of the potassium channels restored cell viability and cell cycle progression when compared to that of wild type cells (**Fig. 1C, 3B** and Fig. S3). Knockout of potassium channel genes exacerbated ZEA-induced cell death due to an impairment of cell cycle progression (**Fig. 3C** and Fig. S4).

To further examine ZEA receptor interactions, the concentration of intracellular and extracellular ZEA toxin by liquid chromatography was analyzed. The quantitative analysis showed that intracellular concentrations were much lower compared to those of extracellular concentrations, with approximately 0.2 to 3.0% at the four incubation times (**Fig. 3D**), suggesting thatit is difficult for the toxin to enter cells. The toxin primarily interacts with the cell membrane proteins in the early stages. However, a small amount of toxin could enter the cell upon disruption of the cell membrane after longer exposure times.

To analyze whether other cytosolic proteins which participate in the regulation of cell cycle progression were involved in ZEA resistance, we engineered the gene *PINK1* to be localized to the cytoplasm for further analysis [17, 18]. Overexpression of *PINK1* failed to provide protection against ZEA, although it induced acceleration of cell cycle progression (**Fig. 3E, F** and Fig. S5). However, *PINK1* deletion significantly influenced cell cycle progression but had no significant effect on ZEA toxicity (**Fig. 3E, F**). Taken together, ZEA caused multiple defects in cell cycle progression due to damaging membrane signaling proteins, such as KCNJ4 and KCNJ12, in the early stages of ZEA exposure.

### Overexpression of potassium channel genes attenuated mitochondrial dysfunction through regulating KCNJ4-mitoKATP pathway in the later stage of ZEA treatment

We further investigated the differential expression profiles following ZEA exposure. The most significantly enriched Gene ontology (GO) and Kyoto Encyclopedia of Genes and Genomes (KEGG) pathways were DNA replication, cell cycle, endoplasmic reticulum (ER), mitochondrial adenosine triphosphate (ATP) synthesis coupling and oxidative phosphorylation in the later stage of ZEA treatment. Compared to wild type cells, in cells overexpressing *KCNJ4* and *KCNJ12*, especially *KCNJ4*, more ion channels were upregulated, which may be responsible for the resistance to ZEA toxicity in later stage (Fig. S6 A, B).

**Figure 4:**
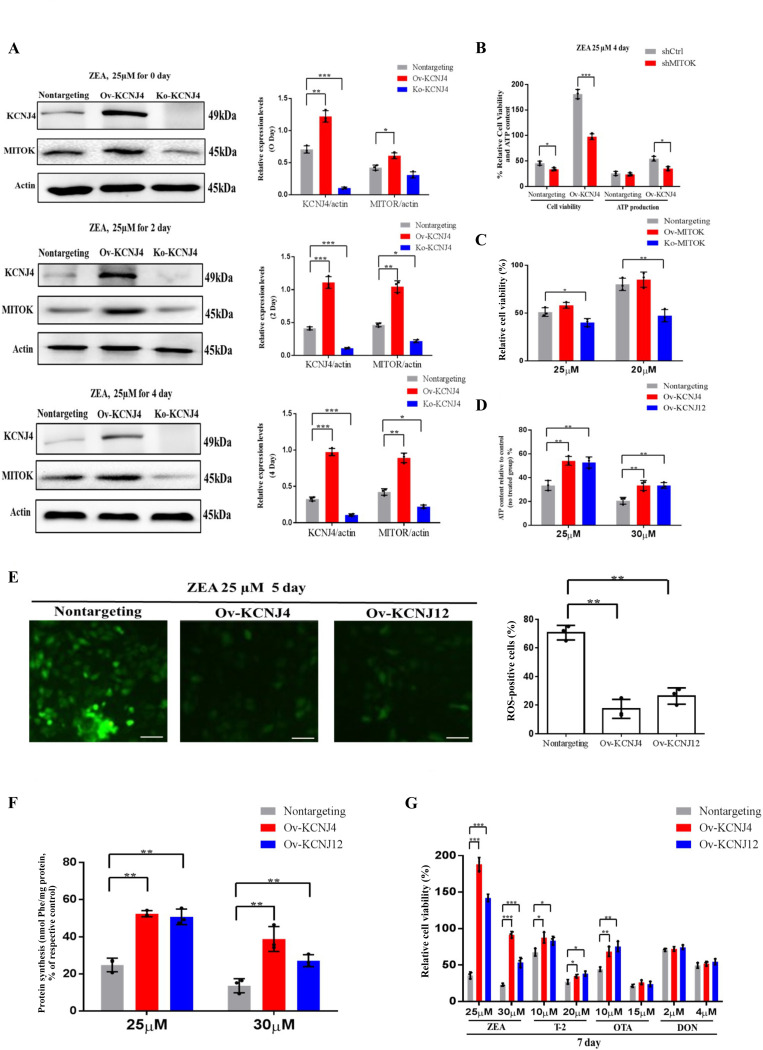
FIGURE 4: High-level potassium channels cell lines attenuate the damage of cellular protein synthesis and mitochondrial under ZEA treatment. **(A)** Immunoblotting for KCNJ4, MITOK and actin expression after ZEA treatment in different times. **(B)** Effect of *MITOK* knockdown on cell viability and ATP production. **(C)** Knockout and overexpression of *MITOK* and the percentage cellular viability of cell lines after treatment with either 25 or 30 µM of ZEA. **(D)** Cellular energy levels measured by ATPlite. **(E)** Cellular ROS levels measured by fluorescence microscopy. **(F)** The levels of protein synthesis were measured following treatment with ZEA for two days. **(G)** The percentage cellular viability of the four cell lines after treatment with various mycotoxins (ZEA, T-2 toxin, OTA, and DON) for 7 days. Data are presented as the mean ± SEM (n=3). p < 0.05 (*), p < 0.01 (**) and p < 0.001 (***), calculated by Student t-test.

When examining the upregulation of ion channels and found that one of the genes, *MITOK*, was 10.4-fold upregulated in overexpressing cells compared to control cells. Further analysis revealed that *MITOK* combined with *MISOUSR* constitutes a mitoK_ATP_ channel, which is a mitochondrial potassium channel [19]. Thus, we inferred that *KCNJ4* could help restore cellular mitochondrial function by activating mitochondrial potassium channels during the later stages of ZEA exposure. Despite mild upregulation of *MITOK* in basal medium, ZEA significantly increased the protein level of the mitoK_ATP_ channel in Ov-KCNJ4 cells, while the loss of *KCNJ4* partially suppressed *MITOK* expression (**Fig. 4A** and Fig. S7, S8 and S9). Furthermore, treatment with 20 nM siRNA for *MITOK* mRNA substantially increased the number of dead cells and decreased ATP production compared to control cells (**Fig. 4B**). Unlike Ov-KCNJ4, overexpression of *MITOK* failed to render cells resistant to ZEA (**Fig. 4C**), and the deletion of *MITOK* aggravated ZEA-induced cell damage as cell viability decreased (**Fig. 4C**). A possible explanation for why activation of *MITOK* did not confer resistance to the ZEA toxin may be that cell injury in the early stage disrupts multiple organelles, while rescuing mitochondria by mediating mitoK_ATP_ channels in later stages was not enough to rescue cell function and survival.

ATP is produced in the mitochondria through oxidative phosphorylation and a direct by-product of ATP production are ROS. Damaged mitochondria produce less ATP and more ROS. Compared with the control group, overexpressing cells showed significantly enhanced ATP production (**Fig. 4D**) and lower levels of intracellular ROS (**Fig. 4E**). Mitochondrial stress causes inefficient translation termination and recycling of mitochondrial outer membrane-associated ribosomes, triggering translation stalling and ribosome-associated quality control [20]. Both edited cells showed greater protein synthesis than control cells (**Fig. 4F**). Altogether, the data indicate that higher expression levels of potassium channels neutralize ZEA toxicity in the later stage by balancing mitochondrial morphology, improving mitochondrial and ribosome function, and attenuating the damage of oxidative stress in the KCNJ4-mitoK_ATP_ pathway.

### Overexpression of potassium channels exhibited resistance to multiple mycotoxins

Apart from the ZEA toxin, ochratoxin A (OTA), trichothecenes (T-2), deoxynivalenol (DON), and other mycotoxins also can induce toxicity and tumorigenesis in both humans and animals [21, 22]. Accordingly, we examined whether overexpression of *KCNJ4* and *KCNJ12* exhibited resistance to other major rodent-related toxins. Indeed, the overexpression cell lines were resistant to T-2 toxin, a trichothecene mycotoxin and byproduct produced by the genus *Fusarium*, at concentrations ranging from 10 to 20 nM. OTA is a mycotoxin produced by *Aspergillus* and *Penicillium* and has been reported to contribute to endemic nephrotoxicity and carcinogenicity in humans and animals [22]. The two cell lines showed higher viability relative to control cells in the presence of OTA, but at a relatively narrow range of OTA concentrations. Regarding the DON toxin there were no significant differences after treatment for seven days. In contrast, these cell lines exhibited more obvious protective effects specific to ZEA (**Fig. 4G**). Overall, the overexpression cells showed lower susceptibility to other mycotoxins, suggesting that mycotoxins have different and distinct toxicity mechanisms.

### *In vivo* recombinant AAV manipulation of potassium channel proteins can attenuate kidney damage

**Figure 5:**
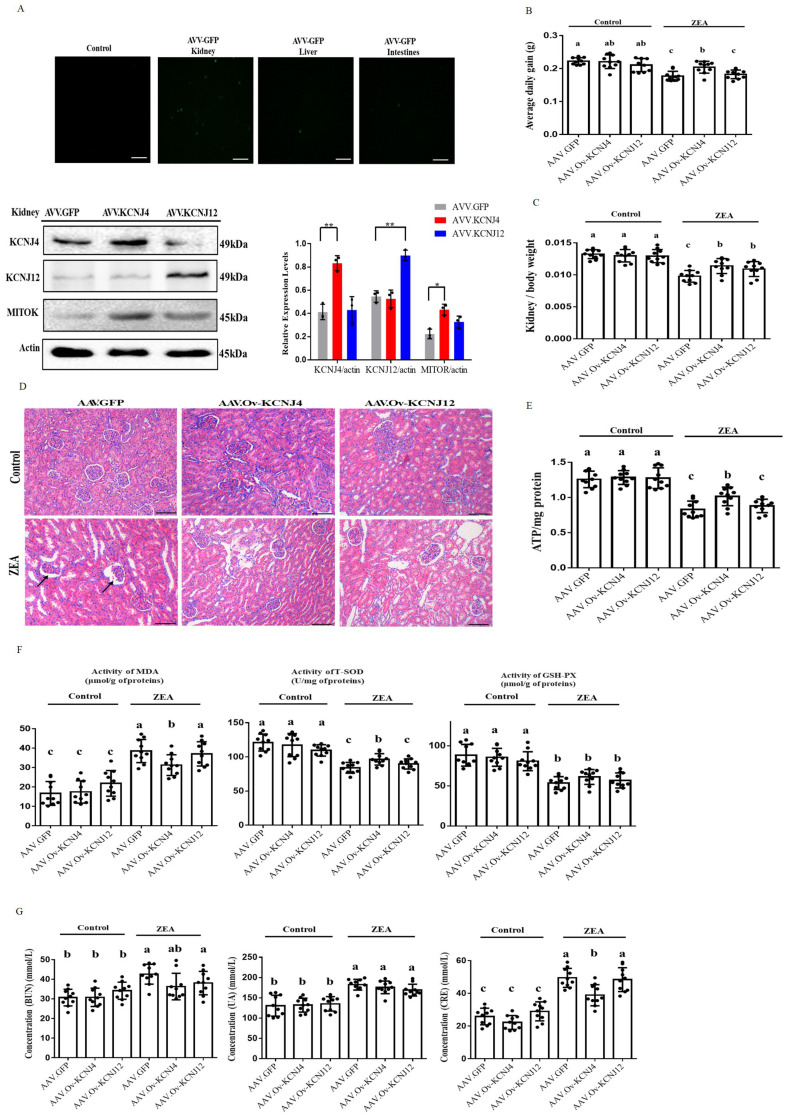
FIGURE 5: *In vivo* AAV manipulation of potassium channel proteins alleviated kidney damage and reduced activities of anti-oxidative enzymes. **(A)** Representative fluorescence photomicrographs of GFP expression in kidneys, live and intestines, and immunoblotting for KCNJ4, KCNJ12, MITOK and actin expression. **(B)** Change in the average daily gain. **(C)** Changes in the ratio of kidney weight to body weight in mice. **(D)** Detection of pathological changes in renal tissue by tissue section HE staining. **(E)** The levels of ATP of renal tissues of mice in each group were detected. **(F)** Oxidation and anti-oxidation parameters of renal tissues of mice in each group were detected by an oxidation kit. **(G)** The content of BUN, UA and CRE in the blood of mice. Animal data of each group are presented as the mean ± SEM (n=10). A two-way ANOVA with Bonferroni posttest was used for Figures B, C, E, F and G. A, B, C Values within a row with different superscripts differ significantly (P < 0.05); Other Data are presented as the mean ± SEM (n=3), calculated by Student t-test. Significant differences were designated as follows: p < 0.05 (*), p < 0.01 (**) and p < 0.001 (***).

To further investigate ZEA resistance *in vivo*, we used recombinant adeno-associated virus (AAV) to study the role of *KCNJ4* and *KCNJ12* in conferring ZEA toxicity in a mouse model. Recombinant AAV is a promising gene delivery vector and has been widely used for genome editing in animals. Using the pAAV-MCS plasmid, we generated recombinant AAVs to determine the function of the potassium channel proteins *in vivo* (Fig. S10). Compared to other tissues, the kidneys were more effectively transduced by AAVs (**Fig. 5A** and Fig. S11). Western blotting confirmed enhanced expression of KCNJ4 and KCNJ12 in the kidneys (**Fig. 5A**). Thus, animal experiments aimed to assess the role of potassium channel proteins in kidney damage. The results show that under ZEA exposure conditions, AAV delivery of the two genes significantly increased the relative weight of the kidney, as well as the body weight gain of the AAV-KCNJ4 mice group compared to the control group (**Fig. 5B, C**). Histopathological analysis showed that ZEA caused lobulation and atrophy of glomeruli in the murine kidney, while the AAV-KCNJ4 group attenuated the pathomorphological changes (**Fig. 5D**). Mechanistically, consistent with the cell changes, *in vivo* expression of *KCNJ4* significantly increased mitochondrial ATP production (**Fig. 5E**), and *MITOK* levels were significantly increased in the kidneys of AAV-KCNJ4 mice compared to that in the control group mice (**Fig. 5A**), suggesting that *KCNJ4* acts as an important factor in maintaining and repairing mitochondrial function.

To confirm the anti-oxidative effect of *KCNJ4* and *KCNJ12* in the kidney [23, 24], we monitored the concentration of total superoxide dismutase (T-SOD), glutathione peroxidase (GSH-Px) and the content of malondialdehyde (MDA), which are markers of lipid peroxidation and antioxidants. Significantly higher levels of T-SOD were found in the AAV-KCNJ4 mice compared to those in the control mice (**Fig. 5F**). Serum activities of blood urea nitrogen (BUN), uric acid (UA), and creatinine (CRE) as biochemical markers are commonly used to assess kidney damage, and the AAV-KCNJ4 group reduced the increase in CRE serum levels compared to those in the control group. (**Fig. 5G**). Collectively, these results indicate that overexpression of *KCNJ4 in vivo* alleviates kidney damage and increases antioxidative enzyme activities by preserving mitochondrial function.

## DISCUSSION

Forward CRISPR-Cas9 activation genetic screening has been developed as a powerful tool for systematic and high-throughput genetic perturbation and provides a phenotype-to-genotype approach for mapping specific genetic perturbations to a phenotype of interest, which has the potential for generating novel therapeutic and regulatory strategies [25–27]. In this study, we found that overexpression of *KCNJ4* and *KCNJ12* could permit cell proliferation in the presence of ZEA and significantly accelerates cell cycle progression, indicating the potential of these two genes to solve mycotoxin problems.

To better understand the effects of toxicity mechanisms at different stages, we identified two genes that have the ability to regulate cell cycle progression {28]. Previous studies have verified that potassium channels are essential in the progression of the cell cycle via their roles in cell permeability and other mechanisms, such as modulation of membrane potential, generation of the driving force in Ca^2+^ transport, and protein-protein interactions. Dysregulation of potassium ion channels significantly influences cell cycle progression [14]. Liquid chromatography data showed that only a small amount of ZEA entered the cells, suggesting that ZEA primarily destroys cell membrane proteins, such as potassium channels, inhibiting cell proliferation and cell cycle progression in the early stage. To further investigate whether other proteins that regulate cell cycle progression are capable of alleviating ZEA toxicity, we demonstrated that editing the cytoplasmic protein PINK1 failed to alleviate ZEA damage, despite altering cell proliferation and cell cycle progression. Combined with the data of intracellular ZEA concentrations and *PINK1* failing to confer ZEA resistance, we propose that ZEA could disrupt the function of potassium channels in the cell membrane, thereby damaging the cells, and increased expression of potassium channels could alleviate this damage to some extent. Thus, our results indicate that the protective mechanism of *KCNJ4* and *KCNJ12* against ZEA is probably through interaction with ZEA, as well as by assisting in regulating cell cycle progression to neutralize toxicity, attenuating the damage of cell cycle arrest, and sustaining cellular homeostasis in the early stages of ZEA exposure.

In the later stages of ZEA exposure, we found that cells experienced more damage of mitochondria, showing persistently high levels of ROS and lower levels of ATP production, which further contributed to the dysfunction of cellular DNA replication, base excision repair, and homologous recombination. The engineered resistant cells acquired tolerance to ZEA, primarily by restoring mitochondrial function in the KCNJ4-mitoKATP pathway.

**Figure 6:**
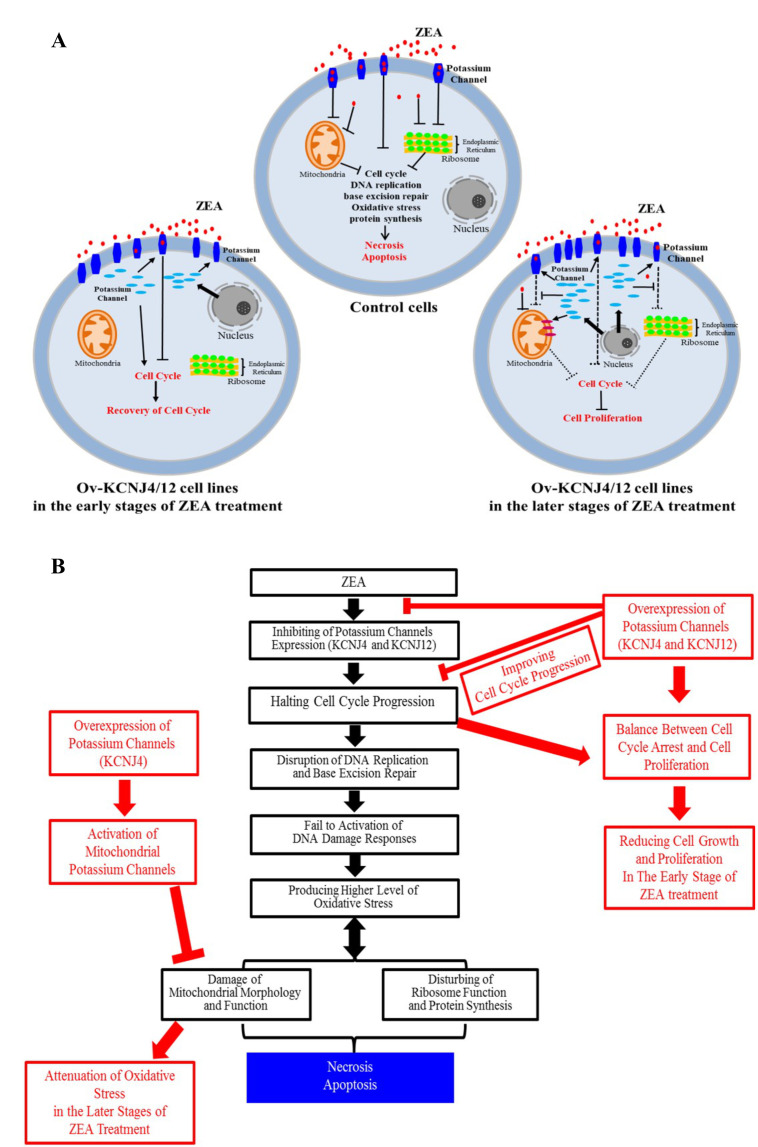
FIGURE 6: Model depicting the interaction between potassium channel genes and ZEA toxin. **(A, B)** Model depicting how the potassium channel genes control for mycotoxin-induced cell death the reacted with ZEA toxin.

Overall, our results indicate that ZEA interacts with the membrane proteins KCNJ4 and KCNJ12 of the target cell, inducing cell cycle arrest, disruption of DNA replication and base excision repair, which causes increased oxidative stress, further injures mitochondria and ribosomes, and halts protein synthesis. Potassium channel proteins, acting as potential targets, were involved in conferring ZEA toxicity by binding and inhibiting the ZEA toxin to improve re-covery of the cell cycle in the early stages of ZEA exposure, as well as sustaining mitochondrial morphology and function, ensuring protein synthesis, and attenuating the damage of oxidative stress in the later stages of ZEA exposure, which substantially reduced cell death (**Fig. 6A, B**). In addition to ZEA, we found that other mycotoxins, such as T2 and OTA, exhibit similar toxicity mechanisms.

In summary, our findings provide important insights into the mechanisms of ZEA toxicity. We observed that cells overexpressing potassium channels can develop resistance to toxins. Edited cells *in vitro* and *in vivo* revealed the underlying mechanisms of the two genes conferring ZEA resistance. We also provide evidence that cell survival is facilitated by potassium channel genes following mycotoxin exposure. The potassium channels KCNJ4 and KCNJ12 are expected to become novel therapeutic targets for mycotoxin treatment and provide effective reference and guidance for the clinical prevention of mycotoxin-induced diseases.

## MATERIALS AND METHODS

### Ethics approval and consent to participate

All animal procedures were conducted according to the Animal Care and Use Committee of the Institute of Subtropical Agriculture, Chinese Academy of Science (IACUC#201302), Date of approval: 21 April 2018.

### Reagents, primer design and cell culture

All primer sequences with clone plasmid and identification can be found in Supplementary Table S1. All cells were grown in high-glucose Dulbecco's modified Eagle's medium (DMEM) containing 10% fetal bovine serum and 1% antibiotic-antimitotic solution (penicillin, streptomycin, and amphotericin B). Cells were tested negative for mycoplasma contamination, but were not authenticated. ZEA was purchased from Sigma (St. Louis, MO, USA) and dissolved in methanol (20 mM). Primary antibodies against KCNJ4 (PA5-102023), KCNJ12 (PA5-55485), MITOK (PA5-52767) and PINK1 (PA5-86941) were purchased from thermos Fisher (Waltham, MA USA), anti-actin (4970) antibodies were purchased from Cell Signaling Technology (Danvers, MA, USA).

### sgRNA library and lentiviral production

The sgRNA library containing activation plasmids (Addgene #1000000078) was amplified and screened according to publish protocols, and lentivirus was produced from HEK-293T cells as described previously [3]. Intestinal HCT-8 and HIEC cells were transduced with a lentivirus-packaged sgRNA library at a multiplicity of infection of 0.3.

### CRISPR-based screening

For generation of a CRISPR-based activation library of cells, 1 × 10^9^ cells were plated onto 150 mm culture dishes to ensure sufficient coverage of sgRNAs. Puromycin (2 μg/mL; Thermo Fisher; A1113803) selection was started at 24 h after lentiviral transduction. The screens were set up until un-transduced control cells were completely dead. For the positive-selection screen, the surviving pool of knockout cells was exposed to 25 µM ZEA for at least 20 day. The remaining cells were then washed and digested to discard attached round-shaped cells and reseeded and cultured with normal DMEM without toxins. Once the screening was complete, genomic DNA was extracted using a Blood and Cell Culture DNA mini kit (Qiagen, Valencia, CA, USA). DNA fragments containing the sgRNAs were amplified, and the abundance of each sgRNA was then analyzed.

### Generation of knockout and overexpression cell line

Guide RNAs were designed using the online CRISPR design tool (http://crispr.mit.edu/) and then cloned into the BsmBI-digested plasmid (lentiCRISPRv2; Addgene #52961) to perform the knockout cell line, or cloned the cDNA into the pLVX plasmid to perform the overexpression cell line according to previously protocol [29, 30].

### Construction of recombinant adeno-associated virus (AAV) for overexpression of mouse potassium channel genes

The plasmid vector (pAVV-KCNJ4 and pAVV-KCNJ12) was constructed by inserting the mouse potassium channel full-length cDNA into the site of pAAV-MCS expression vector for overexpression of the gene. The procedure for constructing the recombinant adeno-associated virus (AAV-2) was performed according to manufacturer's recommended protocol.

### Animal study protocols

All animals used in this study were humanely managed according to the Chinese Guidelines for Animal Welfare. The experimental protocol was approved by the Animal Care and Use Committee of the Chinese Academy of Sciences (Beijing, China) [30]. A total of 60 C57BL/6 mice aged 3-4 weeks were divided into six groups. Viral particles were delivered intravenously via the tail vein at 3 × 10^9^ particles per mouse (0.2 mL). Treatments were as follows: 1. AVV.GFP, 2. AVV-KCNJ4, 3. AVV-KCNJ12, 4. AVV.GFP + ZEA, 5. AVV-KCNJ4 + ZEA, 6. AVV-KCNJ12 + ZEA. The mice in the ZEA group were orally administrated 0.2 mL of PBS containing a 40 mg/kg dose of ZEA every two days and the control group was treated with only PBS [23]. All mice were fed *ad libitum* for four weeks. Body weight gain and food intake were recorded in the experimental period. The blood was collected by blood vessels through the eye socket, bathed at 37°C for 2 h, stood at 4°C for 12 h, centrifuged at 4 000 r/min for 15 min, and the serum was collected into a 1.5 mL centrifuge tube and frozen at -80°C for further determination. After blood collection, samples of kidney, liver and intestines were stored at -80°C for further examination.

### Cell viability assay and siRNAs

Cell Counting Kit-8 (DOJINDO Laboratories, CK04) assay was used to measure cell viability according to the manufacturer's instructions. The relative cell viability was measured relative to viability at timepoint day 0. The cells (1000/well) were seeded into 96 well-plates and were incubated with various treatments as specified for three days. MITOK (sc-78108), siRNAs were purchased from Santa Cruz. Each siRNA product is a pool of three target-specific siRNAs of 19–25 nt. siRNA transfection was performed according to the protocol of the manufacturer. Cells were re-plated at 24 h post-transfection onto a 96-well platen and incubated in arginine-depleted medium for 72 h once they attached.

### Biochemical Assays

Blood samples were centrifuged at 4000 g for 10 min. The serum was collected and used to determine the following biochemical parameters BUN, UA, and CRE. The suspension of the kidney homogenate was collected and assayed for the activities of T-SOD and GSH-Px and the content of MDA. Each measurement was based on the manufacturer's instructions (Nanjing Jiancheng Institute of Bioengineering, China).

### Organ Indexes and HE Staining

All mice were weighed after the last treatment. The organ indexes were obtained from the percentage of kidney wet weight to the total mouse body weight. HE staining was used to examine the pathological changes (three kidney tissues randomly selected from each group were examined).

### Determination of protein synthesis

Protein synthesis was assayed by measuring the incorporation of [3H]-phenylalanine into cellular proteins. After culturing to 90–95% confluence, the cells were incubated for the indicated times in DMEM with ZEA, and the medium was then replaced with DMEM containing [3H]-phenylalanine (0.8 µCi/well, specific activity: 120–190 Ci/mmoL) for 3 h. Cells were washed with PBS three times, and proteins were precipitated by addition of ice-cold 2% (v/v) trichloroacetic acid for 10 min. Proteins were then washed and incubated with methanol for 10 min. The cellular material was solubilized in 1 M NaOH, and incorporation of [3H]-phenylalanine was quantified using liquid scintillation spectrometry.

### Measurement of intracellular ATP levels

ATP levels were determined using an ATPlite 1-step kit (PerkinElmer). Briefly, cells at a density of 5 × 10^3^ cells/well were seeded in 96-well plates and allowed to attach for 4 h. Cells were grown for the indicated times in DMEM with ZEA. Chemiluminescence was measured with an EnVision 2104 Multilabel Reader (PerkinElmer) after adding 100 μL ATPlite solution (Perkin Elmer).

### Identification of GFP expression

Unfixed kidneys were placed in optimal cutting temperature buffer, frozen at −80°C, and cut at 20 μm. Sections were incubated in PBS in a humidified chamber (37°C) for 2 min. GFP expression in kidneys were visualized using Olympus IX73 fluorescence microscopy (Olympus Corporation, Tokyo, Japan).

### Flow cytometry analysis

Following treatment, the cells were collected, and apoptosis was detected by flow cytometry using an AnnexinV-FITC Apoptosis Detection Kit (Beyotime Biotech), following the manufacturer's instructions. Data acquisition and analysis were carried out using a flow cytometry system and related software (Beckman).

### ROS measurement

ROS levels were quantified by staining with dichloro-dihydro-fluorescein diacetate (DCFH-DA; Sigma-Aldrich). Equal numbers of cells were collected after treatment with ZEA and stained with DCFH-DA for 30 min at 37°C. Cells were then washed with phosphate-buffered saline (PBS) and analyzed using a flow cytometer. For each sample, 15,000–20,000 events were recorded. Cells were gated using the forward (FSC) and side (SSC) scatter before assessing fluorescence. Cells with a fluorescence signal above the background, indicating positive for ROS generation, were detected in the FL1 channel (excitation [Ex] at 488 nm and emission [Em] at 530 nm).

### RNA sequencing

Transcriptomic analysis was performed using the strand-specific RNA sequencing protocol described previously^4^. Briefly, total RNA was extracted using an RNeasy Mini kit (Qiagen). Five micrograms of poly (A)-selected RNA were fragmented and dephosphorylated, after which an ssRNA adapter was then ligated. Reverse transcription was performed using a primer complementary to the RNA adaptor, after which a DNA adaptor was ligated onto the 3′ end of the resulting cDNA product. The library was then PCR amplified, cleaned, quantified using a TapeStation (Agilent), and sequenced on a HiSeq 2500 instrument (Illumina). All primer sequences for this protocol were described previously.

### Western blot analysis

All cells were cultured in 6-well plates for the indicated times. Cells were then lysed for 10 min in ice-cold lysis buffer with complete protease inhibitor cocktail. The supernatants were dissolved in Laemmli buffer and separated using a 10% acrylamide-bisacrylamide gel. Proteins were blotted onto a 0.45 μm PVDF membrane. Primary antibody were applied at 1:1000 and Immunoglobulin G (IgG) anti-HRP was diluted 1:5000. The blots were detected using an ECL Plus detection system under conditions recommended by the manufacturer (Thermo). The signals were visualized on Fujifilm LAS-3000 (Tokyo, Japan). Protein band density was normalized to the β-actin signal and quantified using Quantity One software.

### Statistical analysis

Data were expressed as means ± standard errors of the measurement. Differences between groups were analyzed using SPSS 20.0 (SPSS Inc., Chicago, IL, USA). Results with *P* values of at least 0.05 were considered statistically significant.

## SUPPLEMENTAL MATERIAL

Click here for supplemental data file.

All supplemental data for this article are available online at https://www.cell-stress.com/researcharticles/2023a-tang-cell-stress/.

## Abbreviations:

NGS: – next-generation sequencing,
ROS: – reactive oxygen species,
sgRNA: – single guide RNA,
ZEA: – zearalenone.
