# Supplementary material for: CRISPR-activation screen identified potassium channels for protection against mycotoxins through cell cycle progression and mitochondrial function
Source: Cell Stress. 2023 Apr 18;7(5):34–45. doi: 10.15698/cst2023.05.279 (PMC10157994; doi:10.15698/cst2023.05.279)
Supplement: Supplementary file 1 [file ces-07-034-s01.zip › 2023A Tang Cell Stress Supplementals/2023A Tang Cell Stress Supplementary Information.pdf]

Supplementary Information

Supplement Table S1. Primer sequences for sgRNA cloning and validation.

Supplement Figure:

FIGURE S1. (A) Gene ontology enrichment for HCT-8 for 2 day. **(B)** Gene ontology enrichment for HCT-8 for 4 day.

FIGURE S2. The unedited Western Blot images corresponding to Figure 3A

FIGURE S3. The unedited Western Blot images corresponding to Figure 3B

FIGURE S4. The unedited Western Blot images corresponding to Figure 3C

FIGURE S5. The unedited Western Blot images corresponding to Figure 3E

FIGURE S6. A and B) Gene ontology enrichment for overexpression of KCNJ4 and KCNJ12 at 4 days.

FIGURE S7. The unedited Western Blot images corresponding to Figure 4A for 0 day

FIGURE S8. The unedited Western Blot images corresponding to Figure 4A for 2 day

FIGURE S9. The unedited Western Blot images corresponding to Figure 4A for 4 day

FIGURE S10. A) Schematic of three AAV vector.

FIGURE S11. The unedited Western Blot images corresponding to Figure 5A
