# Supplementary material for: CRISPR-activation screen identified potassium channels for protection against mycotoxins through cell cycle progression and mitochondrial function
Source: Cell Stress. 2023 Apr 18;7(5):34–45. doi: 10.15698/cst2023.05.279 (PMC10157994; doi:10.15698/cst2023.05.279)

Figure. S1

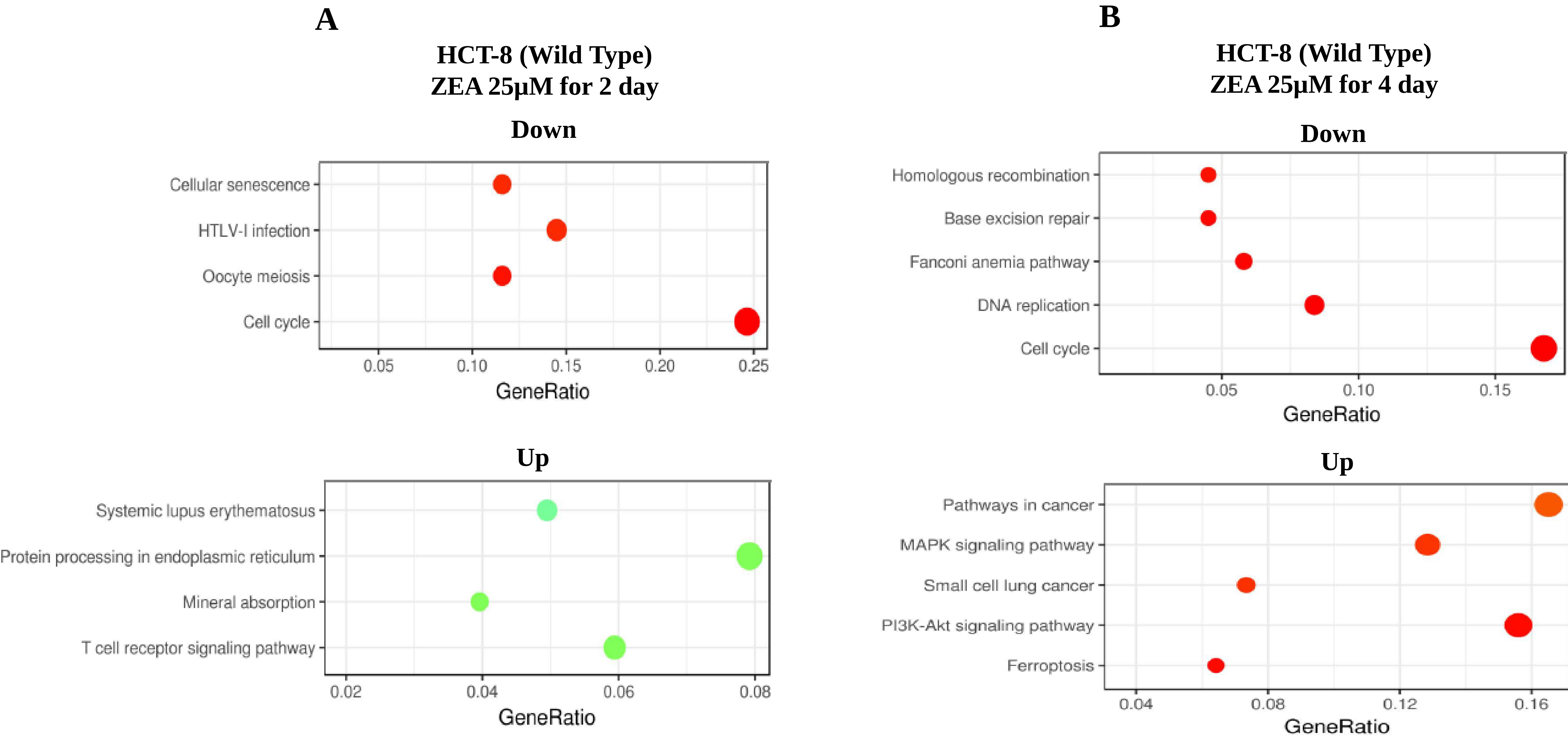
A
HCT-8 (Wild Type)
ZEA 25μM for 2 day
Down
Cellular senescence
HTLV-I infection
Oocyte meiosis
Cell cycle
0.05
0.10
0.15
0.20
0.25
GeneRatio
Up
Systemic lupus erythematosus
Protein processing in endoplasmic reticulum
Mineral absorption
T cell receptor signaling pathway
0.02
0.04
0.06
0.08
GeneRatio
B
HCT-8 (Wild Type)
ZEA 25μM for 4 day
Down
Homologous recombination
Base excision repair
Fanconi anemia pathway
DNA replication
Cell cycle
0.05
0.10
0.15
GeneRatio
Up
Pathways in cancer
MAPK signaling pathway
Small cell lung cancer
PI3K-Akt signaling pathway
Ferroptosis
0.04
0.08
0.12
0.16
GeneRatio

Figure. S2

KCNJ4

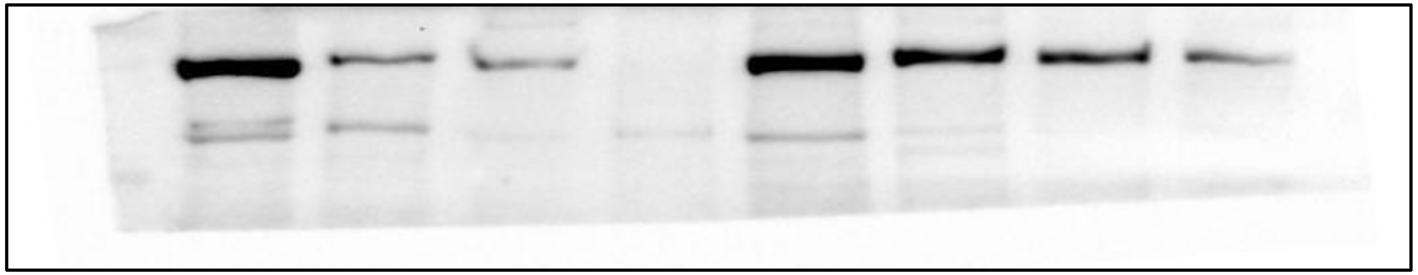

KCNJ12

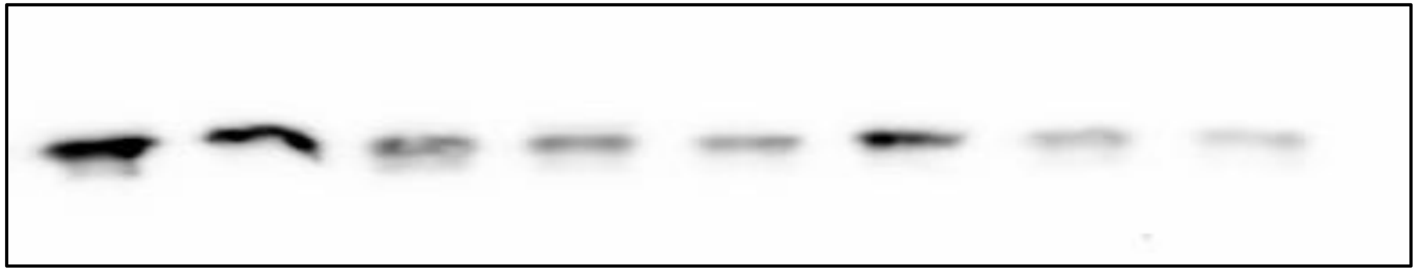

PINK1

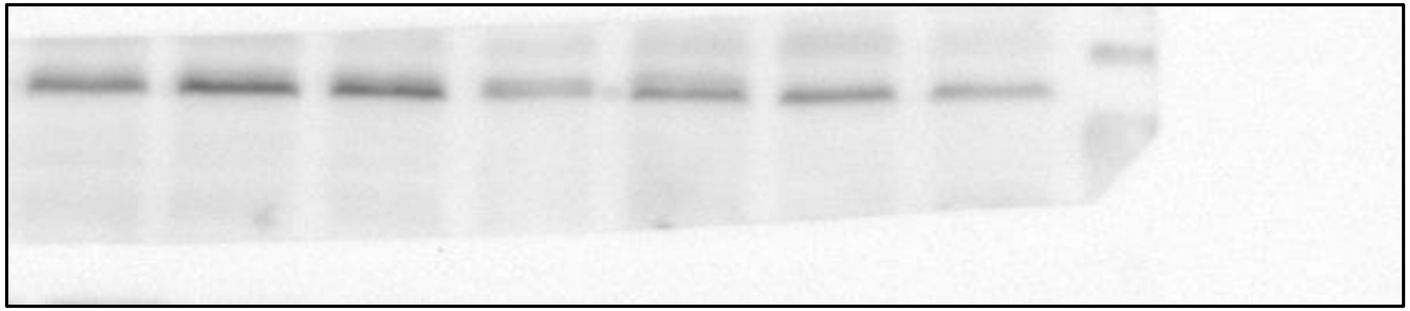

Actin

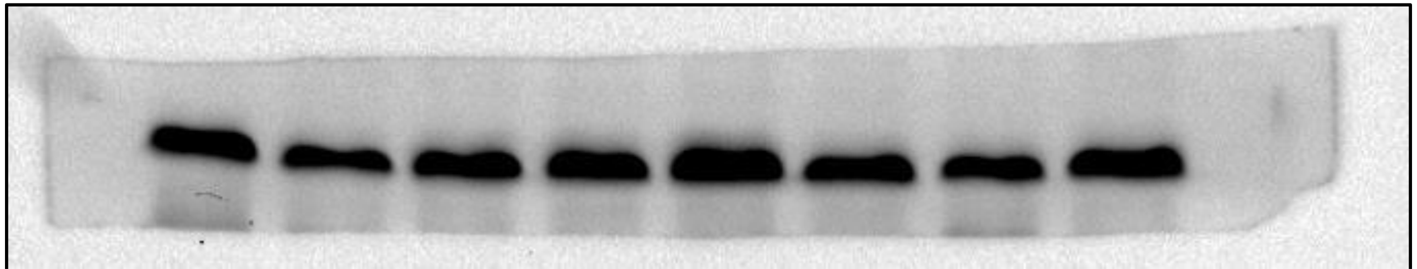

Figure. S3

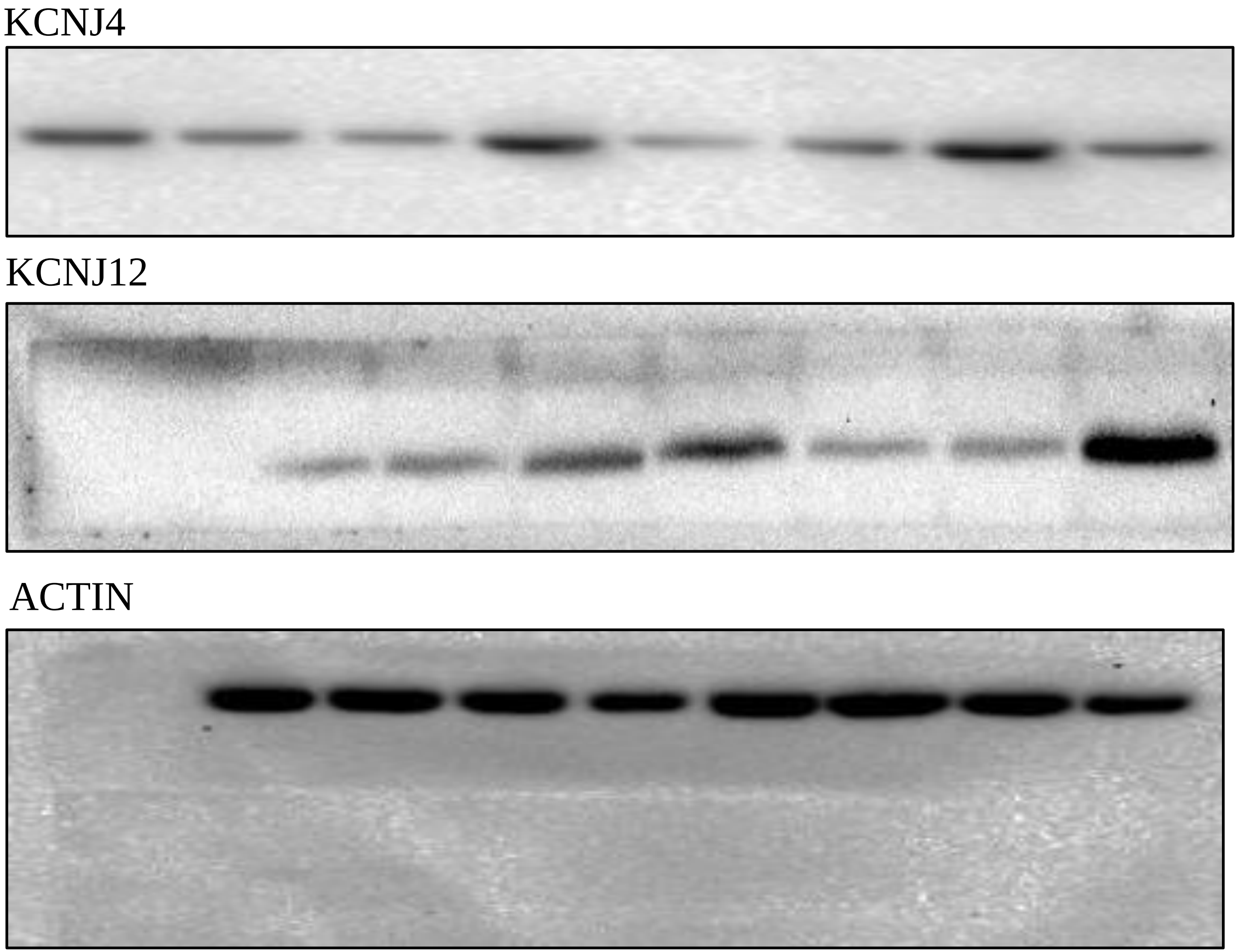

Figure. S4

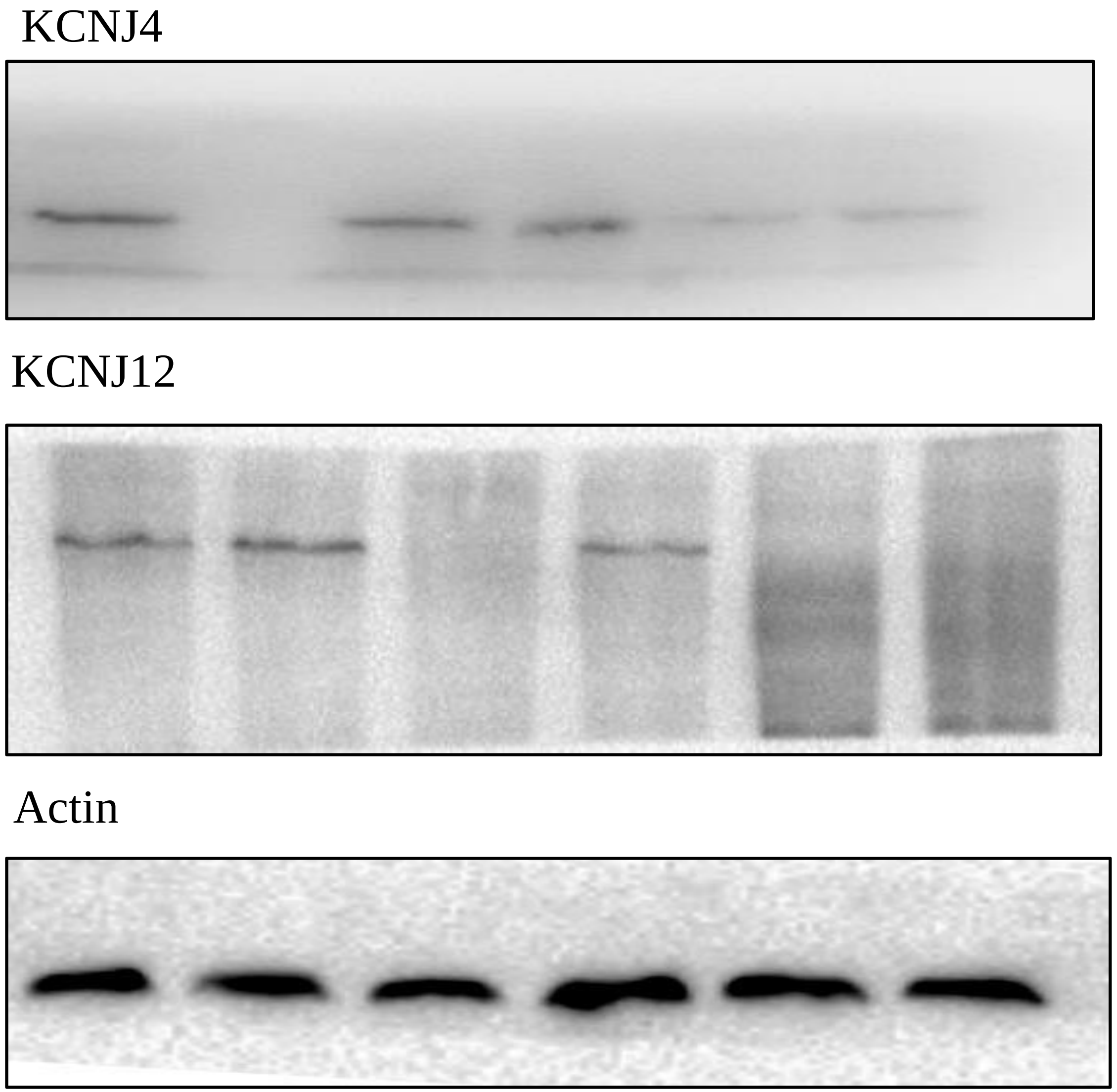

Figure. S5

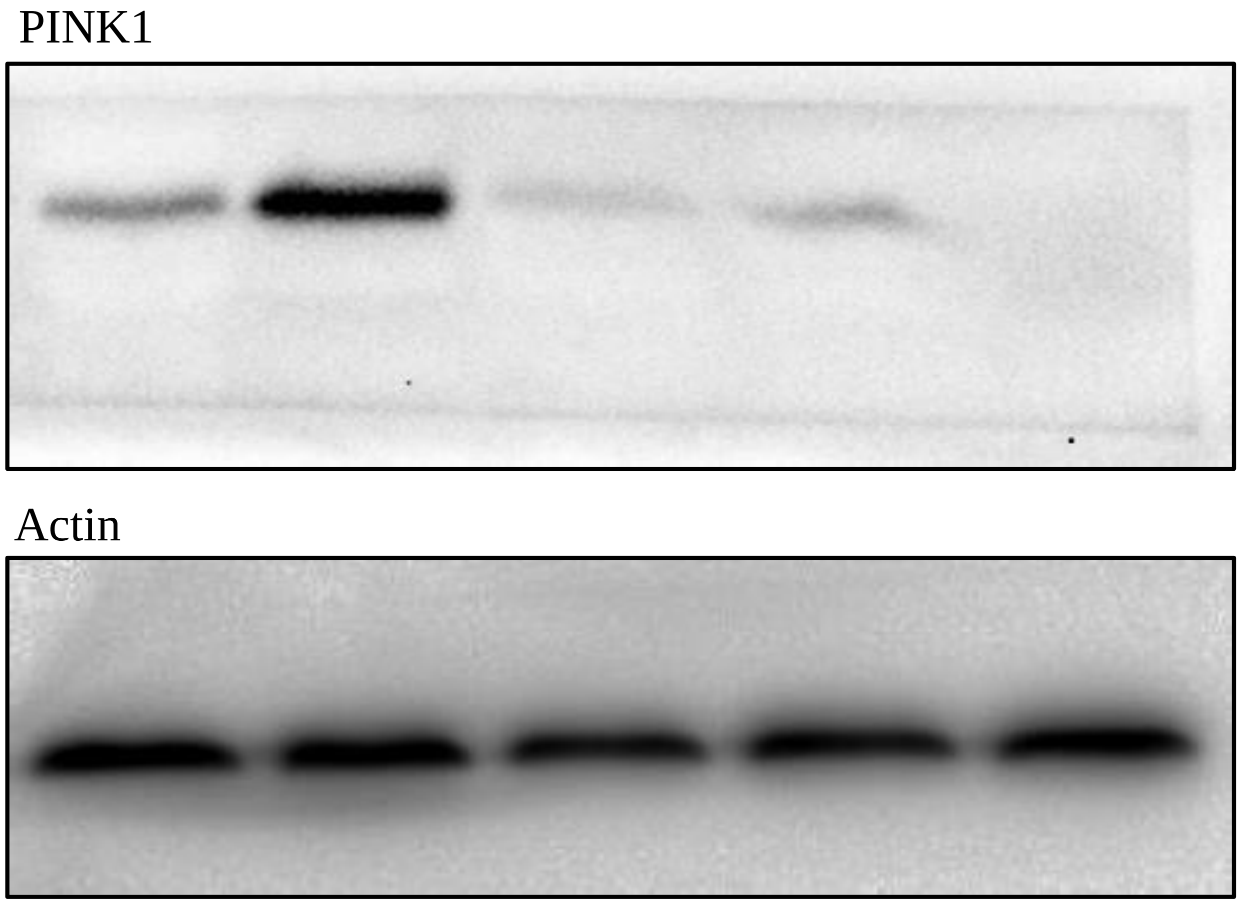

Figure. S6

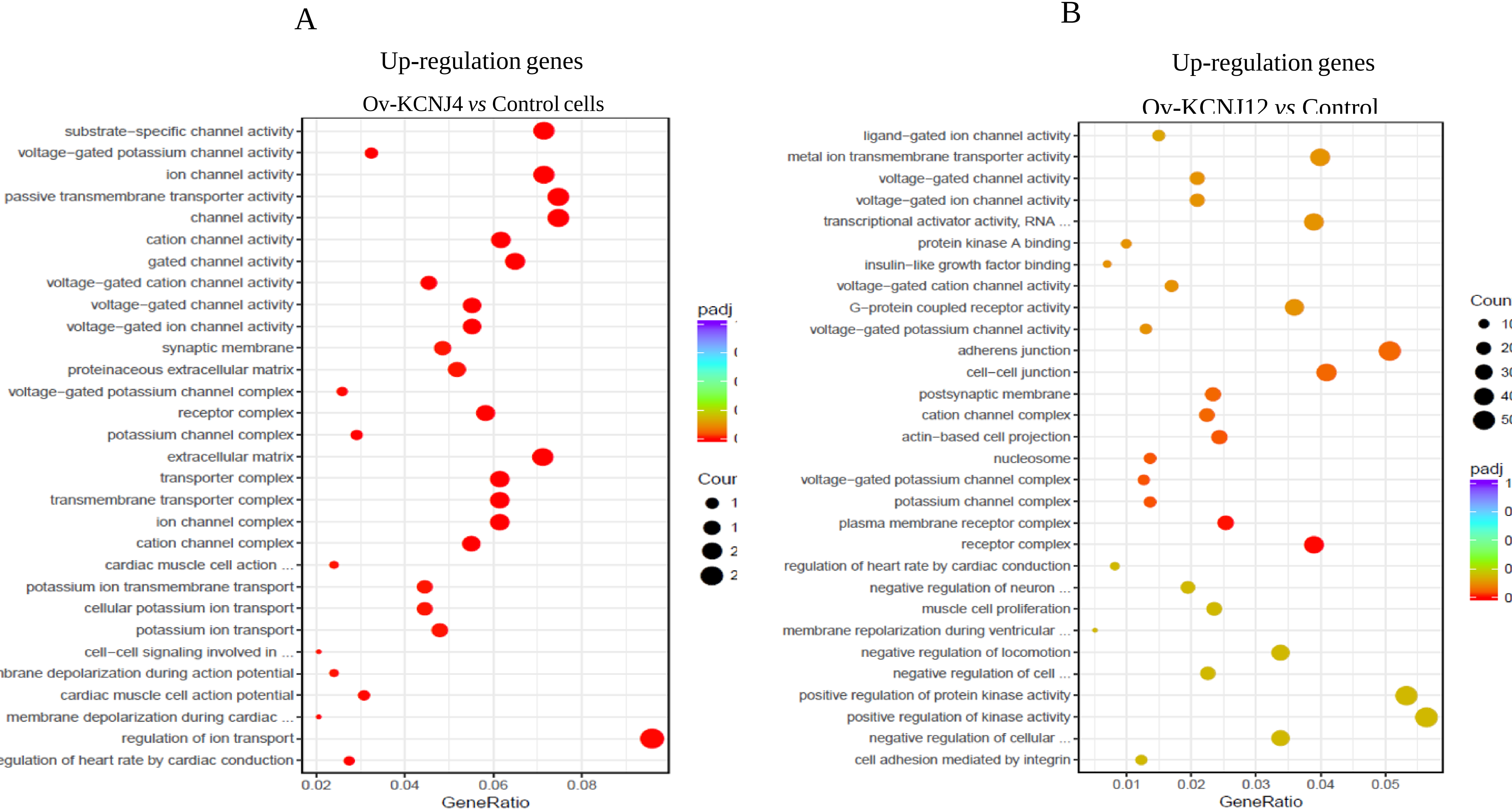
A
Up-regulation genes
Ov-KCNJ4 vs Control cells
substrate−specific channel activity
voltage−gated potassium channel activity
ion channel activity
passive transmembrane transporter activity
channel activity
cation channel activity
gated channel activity
voltage−gated cation channel activity
voltage−gated channel activity
voltage−gated ion channel activity
synaptic membrane
proteinaceous extracellular matrix
voltage−gated potassium channel complex
receptor complex
potassium channel complex
extracellular matrix
transporter complex
transmembrane transporter complex
ion channel complex
cation channel complex
cardiac muscle cell action ...
potassium ion transmembrane transport
cellular potassium ion transport
potassium ion transport
cell−cell signaling involved in ...
brane depolarization during action potential
cardiac muscle cell action potential
membrane depolarization during cardiac ...
regulation of ion transport
egulation of heart rate by cardiac conduction
0.02
0.04
0.06
0.08
GeneRatio
padj
Cour
B
Up-regulation genes
Ov-KCNJ12 vs Control
ligand−gated ion channel activity
metal ion transmembrane transporter activity
voltage−gated channel activity
voltage−gated ion channel activity
transcriptional activator activity, RNA ...
protein kinase A binding
insulin−like growth factor binding
voltage−gated cation channel activity
G−protein coupled receptor activity
voltage−gated potassium channel activity
adherens junction
cell−cell junction
postsynaptic membrane
cation channel complex
actin−based cell projection
nucleosome
voltage−gated potassium channel complex
potassium channel complex
plasma membrane receptor complex
receptor complex
regulation of heart rate by cardiac conduction
negative regulation of neuron ...
muscle cell proliferation
membrane repolarization during ventricular ...
negative regulation of locomotion
negative regulation of cell ...
positive regulation of protein kinase activity
positive regulation of kinase activity
negative regulation of cellular ...
cell adhesion mediated by integrin
0.01
0.02
0.03
0.04
0.05
GeneRatio
Coun
padj

Figure. S7

KCNJ4, 0 day

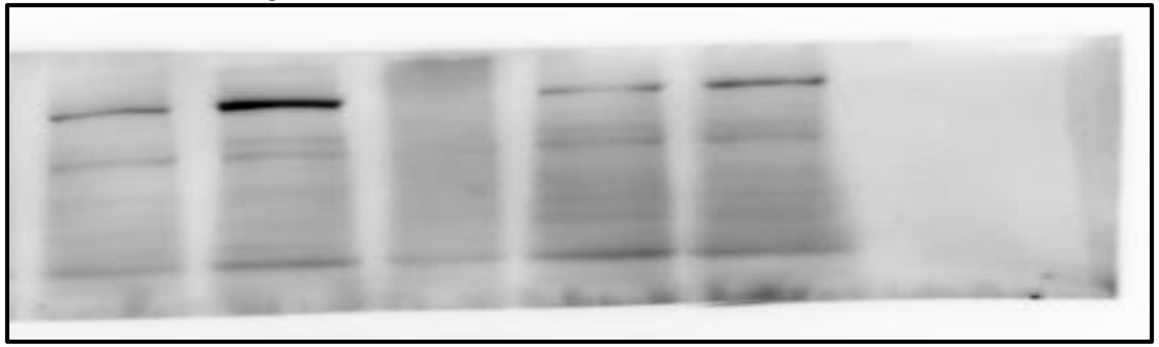

MITOR, 0 day

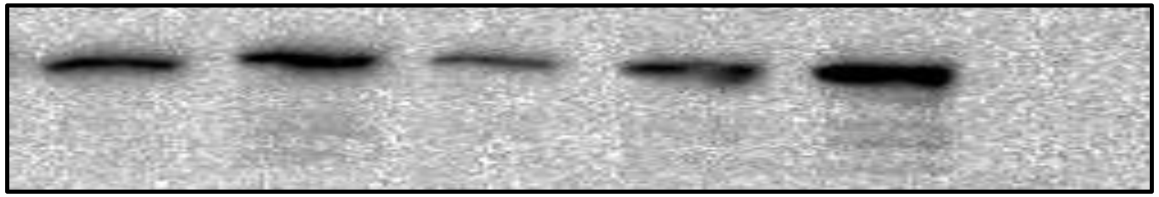

Actin, 0 day

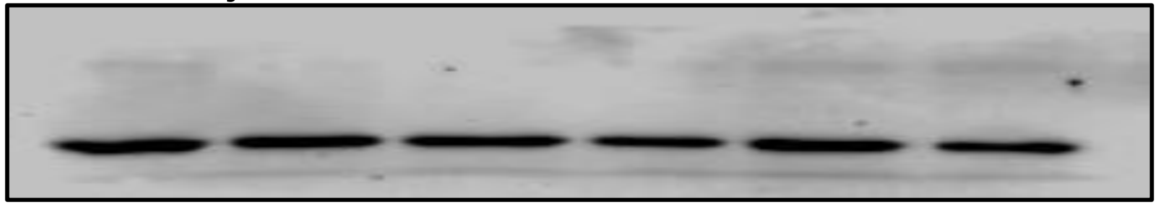

Figure. S8

KCNJ4, 2 day

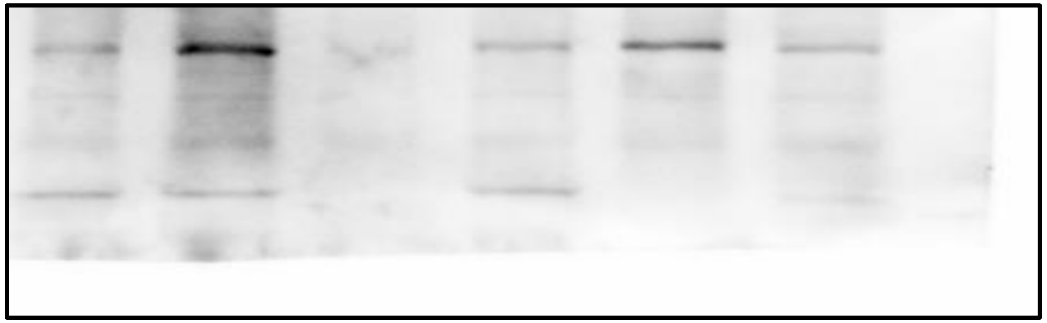

MITOK, 2 day

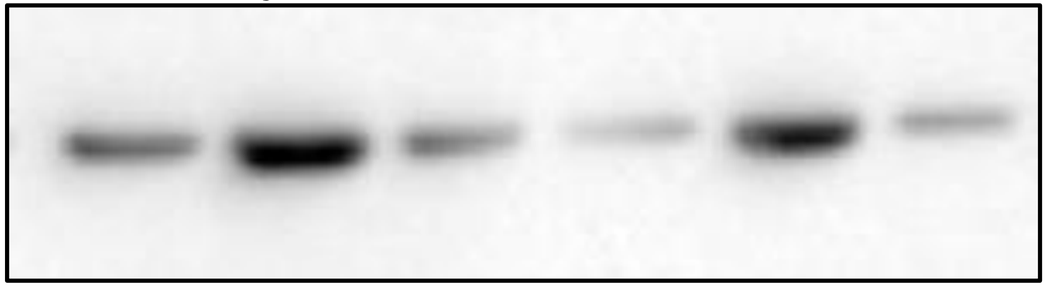

Actin, 2 day

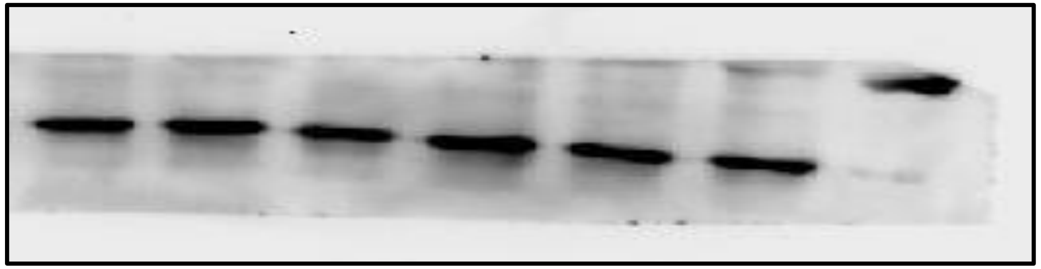

Figure. S9

KCNJ4, 4 day

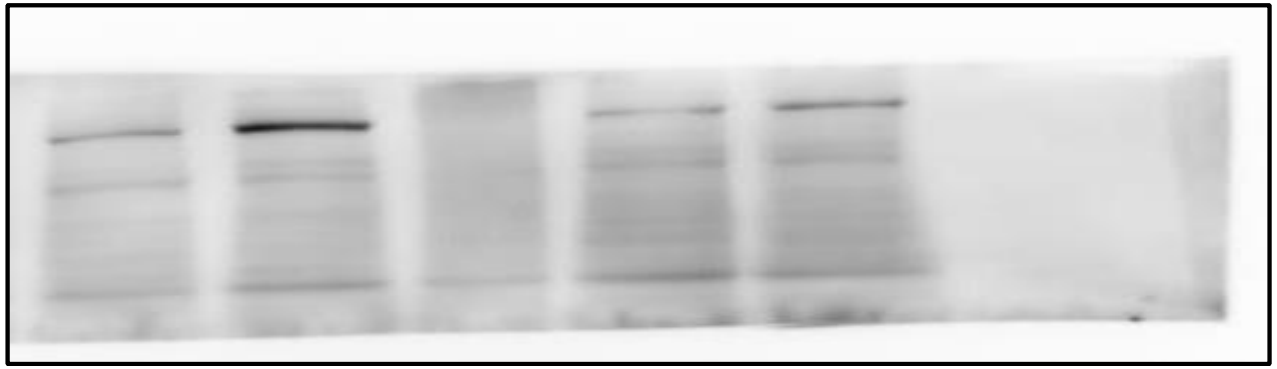

MITOK, 4 day

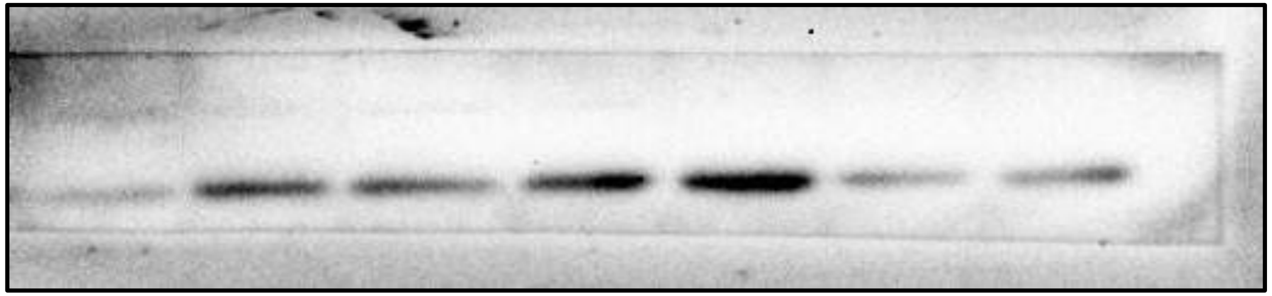

ACTIN, 4 day

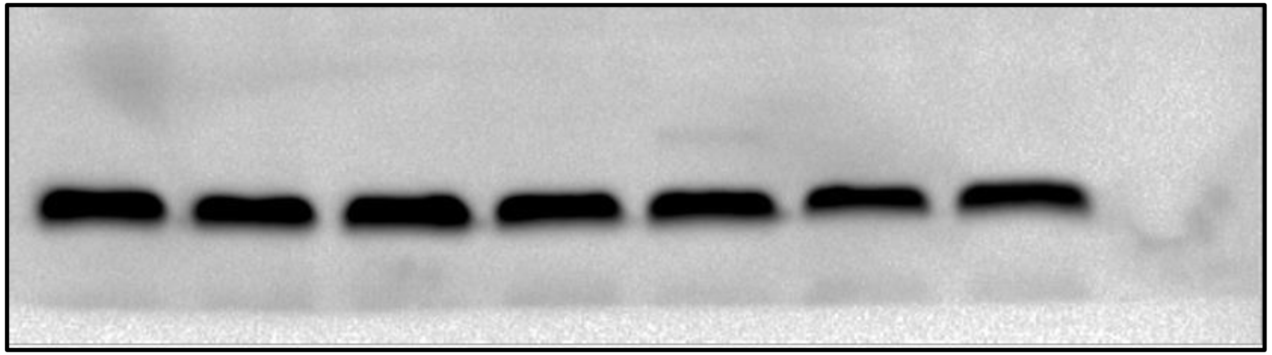

Figure. S10

**A**

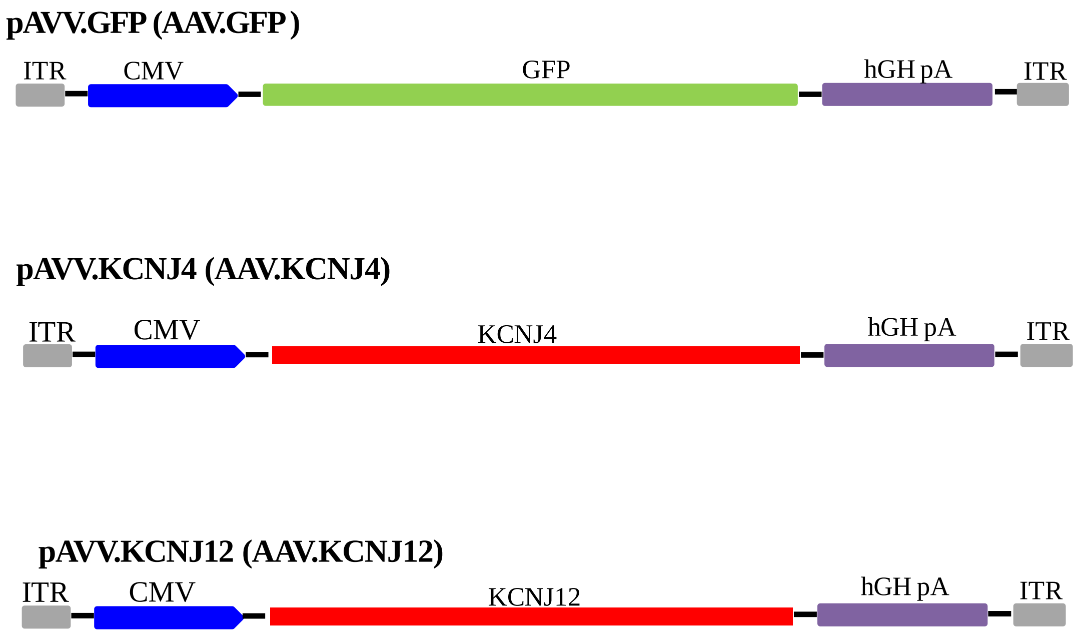
pAVV.GFP (AAV.GFP )
ITR
CMV
GFP
hGH pA
ITR
pAVV.KCNJ4 (AAV.KCNJ4)
ITR
CMV
KCNJ4
hGH pA
ITR
pAVV.KCNJ12 (AAV.KCNJ12)
ITR
CMV
KCNJ12
hGH pA
ITR

Figure. S11

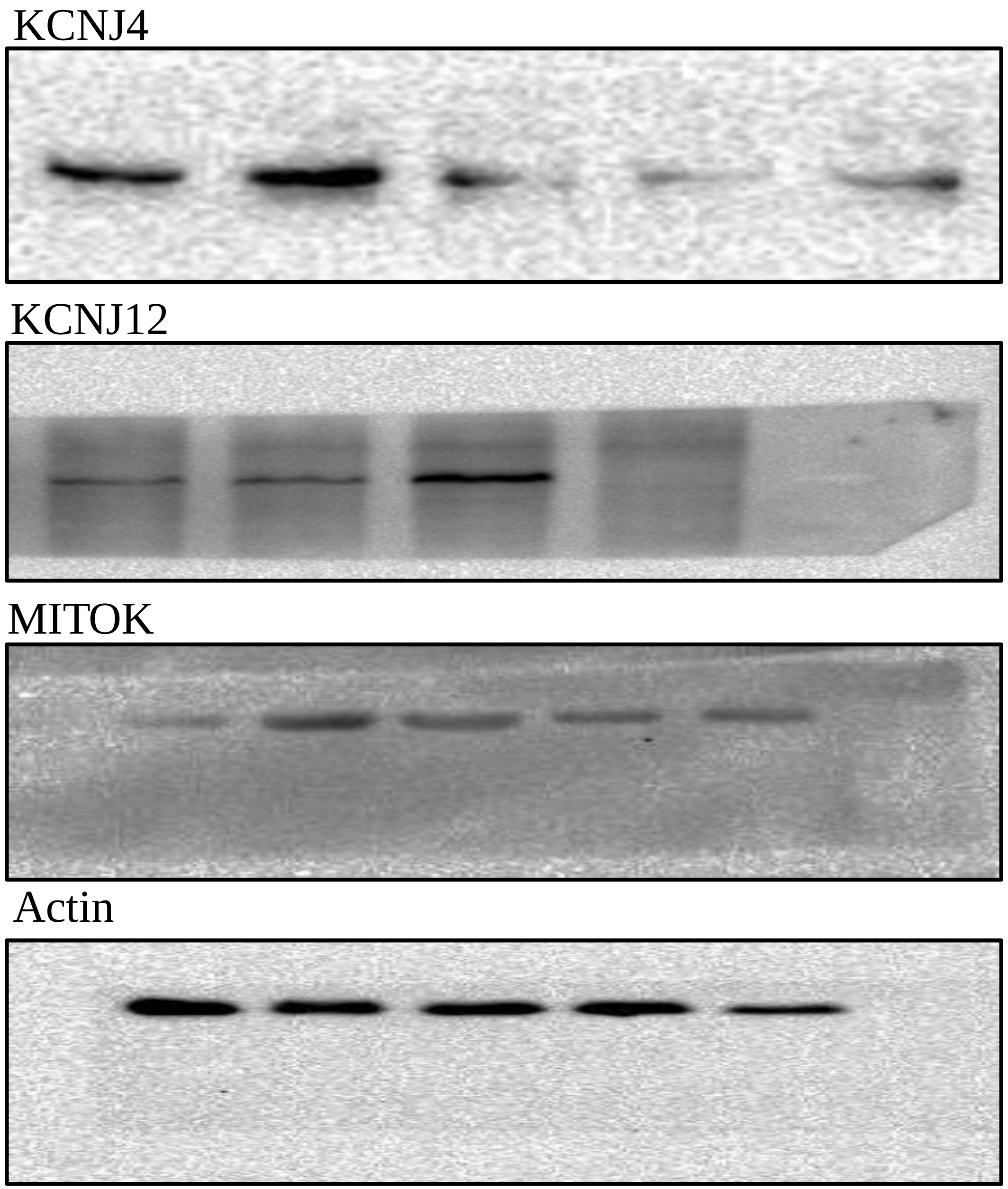

Supplement: Supplementary file 1 [file ces-07-034-s01.zip › 2023A Tang Cell Stress Supplementals/2023A Tang Supplemental Figures.pdf]
